# Preclinical Evaluation of the Assembly Modulator PAV-615 in a Mouse Model of *C9orf72*-Associated ALS/FTD

**DOI:** 10.3390/cells14242012

**Published:** 2025-12-17

**Authors:** Jingfen Su, Jorge Alaiz Noya, Anuradha F. Lingappa, Dennis Solas, Jimei Tong, Lillian Daughrity, Monica Castanedes-Casey, Aishe Kurti, Dennis W. Dickson, Vishwanath R. Lingappa, Leonard Petrucelli, Yongjie Zhang

**Affiliations:** 1 Department of Neuroscience, Mayo Clinic, Jacksonville, FL 32224, USA; su.jingfen@mayo.edu (J.S.); alaiznoya.jorge@mayo.edu (J.A.N.); jimei.tong@gmail.com (J.T.); daughrity.lillian@mayo.edu (L.D.); castanedescasey.monica@mayo.edu (M.C.-C.); kurti.aishe@mayo.edu (A.K.); dickson.dennis@mayo.edu (D.W.D.); petrucelli.leonard@mayo.edu (L.P.); 2 Prosetta Biosciences, Inc., San Francisco, CA 94107, USA; alingappa@prosetta.com (A.F.L.); dsolas@prosetta.com (D.S.); vlingappa@prosetta.com (V.R.L.); 3 Neurobiology of Disease Graduate Program, Mayo Graduate School, Mayo Clinic College of Medicine and Science, Rochester, MN 55902, USA; 4 Department of Physiology, University of California, San Francisco, CA 94158, USA

**Keywords:** assembly modulation, amyotrophic lateral sclerosis, frontotemporal dementia, *C9orf72*, G_4_C_2_ repeat expansion, PAV-615, motor function, anxiety-like behavior, hyperactivity, dipeptide repeat proteins, phosphorylated TDP-43, ataxin 2-positive stress granule

## Abstract

Amyotrophic lateral sclerosis (ALS) and frontotemporal dementia (FTD) are fatal neurodegenerative diseases that share clinical and pathological features, as well as genetic causes. A G_4_C_2_ repeat expansion in chromosome 9 open reading frame 72 (*C9orf72*) is the most common genetic cause of ALS and FTD, collectively referred to as c9ALS/FTD. Assembly modulation is a new therapeutic approach which appears to target allosteric sites on aberrant forms of multi-protein complexes and restore them to the healthy state. Recent findings demonstrate that tetrahydroisoquinolone (THIQ)-based protein assembly modulators can ameliorate ALS/FTD-associated phenotypes in cellular and animal models. In the present study, we investigated the effects of PAV-615, a novel and advanced THIQ-based modulator, in a c9ALS/FTD mouse model expressing 149 G_4_C_2_ repeat expansions (referred to as 149R mouse model). Specifically, PAV-615 was administered to 5-month-old 149R mice via intraperitoneal injection for one month. Motor function was evaluated using the hang wire test, while anxiety-like behavior and hyperactivity were assessed using the open-field test. Pathological markers, including dipeptide repeat (DPR) proteins, phosphorylated TAR DNA-binding protein 43 (pTDP-43) and ataxin 2-positive stress granules, were quantified by Meso Scale Discovery and immunohistochemistry assays. Compared with vehicle-treated controls, PAV-615 significantly improved motor performance and modestly reduced anxiety-like behavior and hyperactivity in 149R mice. Moreover, PAV-615 treatment significantly decreased cortical DPR, pTDP-43 and ataxin 2-positive stress granule burdens. These results support assembly modulation as a promising therapeutic approach treatment of ALS/FTD.

## 1. Introduction

Amyotrophic lateral sclerosis (ALS) is a motor neuron disease marked clinically by progressive muscle weakness and paralysis, typically resulting in death within 3–5 years of symptom onset [1]. Frontotemporal dementia (FTD), the second most common early-onset dementia, is clinically characterized by changes in personality, behavior and/or language, with a mean survival of about 6–8 years following symptom onset [2,3,4]. Despite distinct primary symptoms, ALS and FTD share overlapping clinical, pathological, and genetic features and frequently occur together in the same individuals [5]. The most common genetic cause of ALS/FTD is a GGGGCC (G_4_C_2_) hexanucleotide repeat expansion in the intron 1 of chromosome 9 open reading frame 72 (*C9orf72*), observed in approximately 40% of familial and 12% of sporadic cases [6,7]. These sense G_4_C_2_ repeat expansions undergo repeat-associated non-AUG (RAN) translation to produce toxic dipeptide repeat (DPR) proteins, which include poly(GA), poly(GP), and poly(GR) [8,9,10,11]. Another hallmark of c9ALS/FTD is the presence of neuronal protein aggregates of TAR DNA-binding protein 43 (TDP-43), which is found in approximately 97% of ALS patients and 50% of FTD patients [12,13]. Given the pivotal role of aberrant protein aggregates in disrupting proteostasis in c9ALS/FTD, strategies aimed at reducing protein aggregation and/or restoring proteostasis represent a promising therapeutic approach for treating these fatal neurodegenerative diseases.

Protein assembly modulators are a newly developed class of small molecules that target transient, energy-dependent multiprotein complexes involved in both physiological and pathological protein assembly processes [14]. These compounds were initially identified from a novel phenotypic screen of approximately 150,000 molecules using a cell-free protein synthesis and assembly (CFPSA) system designed to identify inhibitors of host-catalyzed viral capsid assembly without affecting overall protein synthesis [14,15,16,17]. Subsequent studies demonstrated their potential to restore proteostatic homeostasis in models of cancer and neurodegenerative disease [18,19]. These modulators act by interacting with previously unappreciated multiprotein complexes that regulate both pathogenic pathways and homeostatic mechanisms such as p62/SQSTM1, 14-3-3ζ proteins [17] and protein disulfide isomerase (PDI) [18].

Tetrahydroisoquinoline (THIQ) is a privileged structural scaffold that has been incorporated into several clinically used drugs and exhibits broad biological activity [20,21,22,23]. In addition to the known roles in combating infectious diseases, including viral, bacterial, and fungal infections, the THIQ-based compounds have demonstrated neuroactive and neuroprotective effects in central nervous system disorders such as depression [24,25] and Alzheimer’s disease [26,27]. We have developed and tested a series of THIQ-based protein assembly modulators in ALS and FTD models. We observed that these assembly modulators can restore TDP-43 nuclear localization and reduce stress granule formation in patient-derived fibroblasts [28]. Moreover, these assembly modulators also demonstrated efficacy to rescue ALS associated phenotypes in vivo. Specifically, these assembly modulators, rescued motor function in C. elegans expressing ALS-linked A315T TDP-43 mutant, reduced lethality in *Drosophila* expressing (G_4_C_2_)_30_ repeat expansions, and prevented weight loss and neurofilament elevation in superoxide dismutase 1 (SOD1)-G93A mice [28].

To improve the pharmacokinetic properties and safety of the assembly modulator, we performed medicinal chemistry optimization of the THIQ scaffold and developed a new compound, PAV-615. In present study, we evaluated the neuroprotective effects of PAV-615 in a c9ALS/FTD mouse model expressing 149 G_4_C_2_ repeat expansions (referred to as 149R mouse model), which robustly recapitulates the pathological hallmarks and behavioral deficits of c9ALS/FTD [29], thus presenting a valuable mouse model for assessing candidate therapeutics aimed at promoting protein complex disassembly and mitigating neurodegeneration in vivo. We focus on *C9orf72* repeat expansion because it is the most common genetic cause of ALS/FTD and produce DPR and TDP-43 inclusions distinct from the SOD1 pathology observed in ALS patients carrying SOD1 mutations [13]. Moreover, we extended previous findings on THIQ-based assembly modulators by evaluating their ability to reduce TDP-43 aggregates in vivo and further investigated whether these modulators could also alleviate DPR pathology and stress granule-associated inclusions.

## 2. Materials and Methods

### 2.1. Synthesis of PAV-615

PAV-615 was synthesized through the following steps.

Steps 1–5: Generation of 3-Benzyloxy-4-methoxy-acetophenone (step 1), 3-(3-Benzyloxy-4-methoxypheny-1)-2-butenoate (step 2), 3-(3-Benzyloxy-4-methoxyphenyl)-2-butenoic acid (step 3), 3-(3-Benzyloxy-4-methoxyphenyl)-1-nitro-2-butene (step 4), 2-Methyl-2-(3-benzyloxy-4-methoxyphenyl)-l-aminoethane (step 5) has been described in our previous study [28].

Step 6: Generation of (E)-N-[2-(3-benzyloxy-4-methoxy-phenyl)propyl]-3-(6-methylbenzofuran-3-yl)prop-2-enamide: The stirred solution of (E)-3-(6-methylbenzofuran-3-yl)prop-2-enoic acid (step 8) [see synthesis below] (0.68 mM) and 2-(3-benzyloxy-4-methoxy-phenyl)propylamine (step 5) (0.68 mM) in DMF (2 mL) was added HATU (0.82 mM) and diisopropylethylamine (15.0 mM). The reaction mixture was stirred at room temperature for 1 h, then diluted with EtOAc (50 mL), washed sequentially with 10% citric acid and saturated aqueous solution of NaHCO_3_, dried over Na_2_SO_4_, filtered and concentrated to give a residue. The residue was purified by flash chromatography (ethyl acetate/hexanes) to generate compound (E)-N-[2-(3-benzyloxy-4-methoxy-phenyl) propyl]-3-(6-methylbenzofuran-3-yl) prop-2-enamide (80%). MS (*m*/*z*): 456 [M + H].

Step 7: Generation of 6-benzyloxy-7-methoxy-4-methyl-1-[(E)-2-(6-methylbenzofuran-3-yl)vinyl]-1,2,3,4-tetrahydroisoquinoline: A suspension of (E)-N-[2-(3-benzyloxy-4-methoxy-phenyl)propyl]-3-(6-methylbenzofuran-3-yl)prop-2-enamide (step 6, 0.24 mM) in dry acetonitrile (10 mL) was heated under reflux. Phosphorus oxychloride (2.6 mM) was then added dropwise, and the reaction mixture was refluxed for an additional 1 h. The solvent and reagent were removed under vacuum; and the organic layer was washed with water (2 × 10 mL), evaporated in vacuo to give oil, which was then dissolved in ethanol (8 mL), followed by addition of sodium borohydride (0.26 mM). After stirring at room temperature for 30 min, excess reagent was quenched by dropwise addition of 2 M HCl. The reaction mixture was basified with 2 M NaOH and ethanol was evaporated in vacuo to give a residue, which was extracted with water (10 mL) and chloroform (10 mL). The organic phase was washed with water (2 × 10 mL), dried and concentrated to give a residue. The residue was purified by column chromatography (dichloromethane/methanol) to yield 6-benzyloxy-7-methoxy-4-methyl-1-[(E)-2-(6-methylbenzofuran-3-yl) vinyl]-1,2,3,4-tetrahydroisoquinolinetetrahydroisoquinoline (10%). MS (*m*/*z*): 440 [M + H].

Step 8: Generation of (E)-3-(6-methyl1,3-benzodioxol-5-yl)-prop-2-enoic acid: A suspension of 6-methylbenzofuran-3-carbaldehyde (12.2 mM), malonic acid (48.8 mM), pyridine (15 mL), and piperidine (1.22 mM) was heated at 80–85 °C for 1 h and then at reflux (110–115° C) for 3 h. The mixture was diluted with water and subsequently acidified with concentrated HCl. The solution was filtered, and the resulting solid was washed twice with cold water. The residue was dissolved in aqueous NaOH, acidified using aqueous HCl, the precipitated solid was filtered and washed again with cold water to yield the product, (E)-3-(6-methylbenzofuran-3-yl) prop-2-enoic acid, which was used without further purification MS (*m*/*z*): 203 [M + H].

### 2.2. Liquid Chromatography–Tandem Mass Spectrometry (LC-MS/MS) Analysis

LC–MS/MS analysis of PAV-615 was performed by Pharmaron company (Beijing, China). CD1 mice received a single intraperitoneal (IP) injection of PAV-615 at 5 mg/kg. Plasma and brain samples were collected at the following time points (hours): 0.083, 0.25, 0.5, 1, 2, 4, 8, and 24 (n = 3 mice per time point).

To measure PAV-615 concentrations in CD1 mouse plasma, serial working solutions were prepared by diluting the analyte stock with 50% acetonitrile in water. These solutions were spiked into blank plasma to generate calibration standards ranging from (0.5, 1, 2, 5, 10, 50, 100, 500, 1000 ng/mL). Four plasma quality control (QC) samples (1, 2, 50, and 800 ng/mL) were prepared independently from the calibration standards on the day of analysis using the same procedure. These QC samples were prepared on the day of analysis in the same way as calibration standards. Fifteen microliters of each standard, QC samples and unknown samples were added to 200 μL of acetonitrile containing internal standard (IS) mixture for precipitating protein, respectively. Then the samples were vortexed for 30 s. After centrifugation at 4 °C, 4000 rpm for 15 min, the supernatant was diluted 3 times with water. Twenty microliters of diluted supernatant were injected into the LC/MS/MS system for quantitative analysis.

To measure PAV-615 concentrations in CD1 mouse brain, brain tissues were homogenized in water at a weight-to-volume ratio of 1:4 (g:mL). Serial working solutions were prepared by diluting the analyte stock with 50% acetonitrile in water, then spiked into blank CD1 mouse brain homogenate to generate calibration standards ranging from 0.5 to 1000 ng/mL (0.5, 1, 2, 5, 10, 50, 100, 500, 1000 ng/mL). Four independently prepared quality control (QC) samples (1, 2, 50, and 800 ng/mL) were freshly made on the day of analysis using the same procedure. For extraction, 45 μL of each standard, QC, or test sample was mixed with 200 μL of IS solution to precipitate proteins, vortexed for 30 s, and centrifuged at 4 °C, 4000 rpm for 15 min. The resulting supernatant was diluted threefold with water, and 20 μL was injected into the LC–MS/MS system for quantitative analysis.

### 2.3. Animal Studies

A total of 24 male CD1 mice were used for pharmacokinetic studies of PAV-615. The research was performed according to Animal care and Use Application (AUP) approved by the Institutional Animal Care and Use Committee (IACUC) of Pharmaron following the guidance of the Association for Assessment and Accreditation of Laboratory Animal Care (AAALAC). The procedures were approved by the Pharmaron Institutional Animal Care and Use Committee (IACUC) (Protocol numbers, PK-M-07182024).

A total of 56 C57BL/6J mice (Jackson Laboratory, Bar Harbor, ME, USA) were used for PAV-615 administration, behavior tests and biochemical analysis, including 31 males and 25 females. All procedures complied with the National Institutes of Health Guide for the Care and Use of Laboratory Animals. The procedures were approved by the Mayo Clinic IACUC (Protocol numbers, A00007238-23).

All mice were maintained under a 12 h light/dark cycle with free access to standard chow and water.

### 2.4. Virus Production

rAAV9 viruses were produced as previously described [29]. AAV vectors encoding (G_4_C_2_)_2_, or (G_4_C_2_)_149_ were co-transfected with helper plasmids into HEK293T cells using polyethylenimine (Polysciences, Inc., Warrington, PA, USA, #23966). Forty-eight hours post-transfection, cells were harvested and lysed by freeze-thawing in the presence of 0.5% sodium deoxycholate and 50 U/mL Benzonase (Millipore Sigma, Darmstadt, Germany, #1016950001). Viral particles were purified using a discontinuous iodixanol gradient. Genomic titers were quantified by qRT-PCR. Final viral stocks were diluted in sterile 1× Dulbecco’s phosphate-buffered saline (DPBS) (Thermo Fisher Scientific, Waltham, MA, USA, #14190144).

### 2.5. Neonatal Viral Injections

Intracerebroventricular injections were conducted on postnatal day 0 (P0) C57BL/6J mouse pups as previously described [29]. In brief, 2 µL of rAAV solution containing rAAV9-(G_4_C_2_)_2_ (0.75 × 10^10^ genomes/µL), or rAAV9-(G_4_C_2_)_149_ (2.5 × 10^10^ genomes/µL) were delivered into each lateral ventricle of cryo-anesthetized pups. Following injection, pups were warmed on a heating pad for recovery before being returned to their home cage with the dam.

### 2.6. Intraperitoneal Injection of PAV-615 or Vehicle

Five-month-old male and female mice were administered the small molecule PAV-615 (Prosetta Biosciences, San Francisco, CA, USA) or vehicle via IP injection. Each treatment group of 2R mice (PAV-615 or vehicle) included 13 animals, and each treatment group of 149R mice included 15 animals. The samples size was determined based on our previous publication [29].

PAV-615 was dissolved in vehicle composed of 10% DMSO, 40% PEG-400, and 50% sterile saline, freshly prepared prior to each injection. The final working solution was filtered through a 0.22 μm syringe filter to ensure sterility.

Mice were gently restrained without anesthesia and IP injected with PAV-615 (2.5 mg/mL) at a dose of 10 mg/kg body weight using a 1 mL insulin syringe with a 27G needle. Injections were performed in the lower right abdominal quadrant to avoid injury to internal organs. Mice that received the vehicle were used as control groups.

Injections were administered once daily for 30 consecutive days, during the light cycle (between 9 and 11 a.m.), and animals were monitored for signs of distress or weight loss throughout the treatment period. All procedures were performed in accordance with institutional IACUC guidelines and approved animal use protocols.

### 2.7. Behavioral Testing

Six-month-old rAAV9-(G_4_C_2_)_2_ (n = 26) or rAAV9-(G_4_C_2_)_149_ (n = 30) mice underwent behavioral assessments over two consecutive days at the Mayo Mouse Behavior Core. On day 1, mice were tested for open-field test, and on day 2 for hanging wire test. All mice were acclimated to the testing room for 1 h before each session and returned to their home cages afterward.

The open-field test was performed as described previously [29]. Briefly, mice were individually placed in a square arena, and their activity was recorded for 15 min using an automated tracking system. Locomotor data was analyzed with Anymaze software 7.51 (Stoelting Co., Wood Dale, IL, USA).

The hanging wire test was performed as previously described [29]. In brief, the apparatus consisted of a 2 mm thick wire (55 cm wide) suspended 35 cm above a padded bedding surface. Mice were gently lifted by the tail and allowed to grasp the wire with their forelimbs for up to 2 min. Falls were recorded, and mice were immediately placed back on the wire after each fall.

### 2.8. Tissue Processing

Mice were euthanized by CO_2_ inhalation and brains were rapidly harvested and bisected sagittally. One hemibrain was fixed in 4% paraformaldehyde, embedded in paraffin, sectioned at 5 μm, and mounted on glass slides for immunohistochemistry. The contralateral hemibrain was dissected into specific regions (cortex, hippocampus, cerebellum, other tissues) and snap-frozen on dry ice. For biochemical analyses, frozen cortical tissue was homogenized in ice-cold TBSE lysis buffer (50 mM Tris-HCl pH 7.4, 50 mM NaCl, 1 mM EDTA) supplemented with protease and phosphatase inhibitors (50×), and the resulting lysates were collected for protein extraction.

### 2.9. Preparation of Brain Protein Lysates

Protein lysates were prepared by supplementing tissue homogenates with Triton X-100 and 10% sodium dodecyl sulfate (SDS) to reach final concentration of 1% and 2%, respectively. The mixtures were then sonicated on ice to ensure complete cell disruption and centrifuged at 16,000× *g* for 20 min. Clear supernatants were collected for analysis, and total protein levels were quantified using the Bicinchoninic Acid (BCA) assay (Thermo Fisher Scientific, 23227).

### 2.10. Immunohistochemistry (IHC)

Sagittal paraffin-embedded brain sections (5 μm) were mounted on positively charged slides, dried overnight, and processed for IHC. Slides were deparaffinized in xylene, rehydrated through graded ethanol, and rinsed in distilled water. Antigen retrieval was performed by steaming in 10 mM sodium citrate buffer (pH 6.0, 0.05% Tween-20) for 30 min. After cooling, sections were treated with Dako Peroxidase Block (Agilent Technologies, S2001) for 5 min to quench endogenous peroxidase activity, followed by a 1 h blocking with Dako Protein Block Serum-Free (Agilent Technologies, Santa Clara, CA, USA, #X0909) for DPR staining or 2% normal goat serum for pTDP-43 staining.

For DPR staining, tissue sections were immunostained with primary antibodies (Table S1) using the Thermo Scientific Autostainer 480S along with the Dako EnVision + System-HRP Labelled Polymer (Agilent Technologies, Santa Clara, CA, USA). Primary antibodies (listed in Table S1) were applied for 45 min, followed by washes and a 30 min incubation in Dako Envision-Plus anti-rabbit (Agilent Technologies, K4003) labeled HRP polymer. Signal development was achieved using the Liquid DAB + Substrate Chromogen System (Agilent Technologies, K3468).

For pTDP-43 staining, tissue sections were incubated overnight at 4 °C with the primary antibody listed in Table S1. After washing in PBS, slides were treated for 2 h with a biotinylated secondary antibody (1:200) and then exposed to an avidin-biotin complex (ABC) solution (Vector Labs, Newark, CA, USA) for 30 min. The peroxidase reaction was developed using 3,3′-Diaminobenzidine chromogen substrate (Acros Organics, Geel, Belgium) with hydrogen peroxide.

All sections were counterstained with hematoxylin (Thermo Fisher Scientific), dehydrated through graded ethanol, cleared in xylene, and mounted with Cytoseal mounting medium (Thermo Fisher Scientific).

### 2.11. Quantification of Neuropathology

To quantify DPR and pTDP-43 burden, high-resolution digital images of immunostained sections were acquired using a ScanScope AT2 (Leica Biosystems, Wetzlar, Germany). The percentage area of DPR [poly(GA), poly(GP), or poly(GR)] immunoreactivity was measured with ImageScope software (v12.4, Leica Biosystems) as previously described [29]. The entire cortical region was annotated on mid-sagittal sections, and the ratio of positively stained pixels to total pixels was calculated to determine DPR burden. Cortical pTDP-43 inclusions were manually counted within the same regions.

### 2.12. Meso Scale Discovery (MSD) Immunoassay

MSD sandwich assays were conducted as previously reported [30]. In brief, equal amounts of total protein from mouse cortex were diluted in Tris-buffered saline (TBS) or Diluent 100 (MSD, Rockville, MD, USA, # R50AA-2) and analyzed using custom MSD-based sandwich immunoassays for poly(GR), poly(GA), or poly(GP). The detailed information for DPR MSD assay is listed in Table S2. Electrochemiluminescent signals were acquired by the MSD QUICKPLEX SQ120 system (MSD, Rockville, MD, USA). Background was corrected using signal values from negative controls (lysates from control mice).

### 2.13. Statistics

Data are presented as mean ± SEM and analyzed using two-tailed unpaired *t*-tests or one-/two-way ANOVA with Tukey’s post hoc test (GraphPad Prism 10.4.1, GraphPad Software, San Diego, CA, USA). Statistical significance was defined as *p* < 0.05. Assumptions of statistical tests were not formally assessed. Additional methodological information is available in the respective figure legends.

## 3. Results

### 3.1. PAV-615 Improves Behavioral Performance in the 149R ALS/FTD Mouse Model

**Figure 1:**
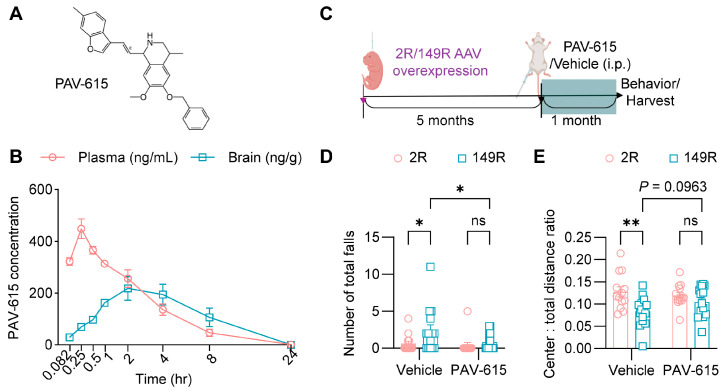
PAV-615 treatment improves behavioral performance in the 149R ALS/FTD mouse model. (**A**) Structure of PAV-615; (**B**) Time-course plot of PAV-615 concentrations in plasma (ng/mL) and brain (ng/g) following a single intraperitoneal (IP) dose administered to CD1 mice (n = 3 per time point). (**C**) Schematic of the experimental paradigm for PAV-615 administration in 149R and 2R mice. (**D**) Hanging wire test assessing motor function by counting the number of falls in 6-month-old 2R and 149R mice treated with vehicle or PAV-615 (n = 13–15 per group). (**E**) Open field assay assessing anxiety by evaluating the ratio of distance traveled in the center area to total distance traveled in 6-month-old 2R and 149R mice treated with vehicle or compound (n = 13–15 per group). Data shown as the mean ± SEM. ns: not significant, * *p* < 0.05, ** *p* < 0.01, two-way ANOVA followed by multiple-comparison test.

By medicinal chemistry modification on the THIQ scaffold (Figure S1), we obtained a novel compound PAV-615 (Figure 1A). Pharmacokinetic analysis demonstrated that PAV-615 was rapidly absorbed following a single intraperitoneal (IP) injection (5 mg/kg) in CD1 mice (Figure 1B). In plasma, PAV-615 was detectable within 5 min, reached peak concentration at approximately 15 min, and subsequently declined in a time-dependent manner, becoming undetectable by 24 h. In the brain, PAV-615 was also detected within 5 min, reached peak levels at approximately 2 h, and subsequently declined to undetectable levels by 24 h post-administration (Figure 1B). These data indicate that PAV-615 exhibits rapid brain penetration but a relatively short duration of exposure (<24 h). Based on these findings, a daily 10 mg/kg dosing regimen was selected for subsequent efficacy studies in the c9ALS/FTD mouse model.

To investigate the effect of PAV-615 in c9ALS/FTD, we generated a cohort of (G_4_C_2_)_149_ (149R) and (G_4_C_2_)_2_ (2R) mice by intracerebroventricular (ICV) injection of AAVs into the lateral ventricles of P0 mice. At 5 months of age, mice were treated with PAV-615 at 10 mg/kg or vehicle via daily IP injection for four weeks (Figure 1C). During the final week of treatment, the hang wire test and open field assay were conducted to evaluate whether PAV-615 ameliorates behavioral deficits in 149R mice. In the hang wire test, vehicle-treated 149R displayed motor deficits, as indicated by a higher number of falls compared to vehicle-treated 2R control mice (Figure 1D). In the open filed assay, vehicle-treated 149R exhibited a decreased tendency to explore the center of the open field and traveled a greater distance compared to vehicle-treated 2R control mice (Figure 1E and Figure S2), indicating exhibit anxiety-like behavior and hyperactivity, respectively. Notably, treatment with PAV-615 significantly improved motor performance, as evidenced by fewer total falls (Figure 1D), and modestly alleviated anxiety-like behavior and hyperactivity, as shown by increased center exploration (Figure 1E) and decreased total travel distance (Figure S2). Together, these findings indicate that PAV-615 treatment mitigates behavioral deficits in 149R mice.

### 3.2. PAV-615 Does Not Affect Body and Brain Weight in the 149R ALS/FTD Mouse Model

**Figure 2:**
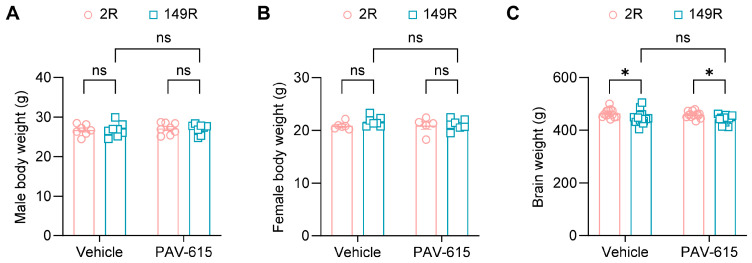
PAV-615 treatment does not affect body and brain weight in the 149R ALS/FTD mouse model. (**A**) Mean body weight of 6-month-old male 2R and 149R mice treated with vehicle or PAV-615 (n = 7–8 per group). (**B**) Mean body weight of 6-month-old female 2R and 149R mice treated with vehicle or PAV-615 (n = 5–7 per group). (**C**) Mean brain weight of 6-month-old 2R and 149R mice treated with vehicle or PAV-615 (n = 13–15 per group). Data shown as the mean ± SEM. ns: not significant, * *p* < 0.05, two-way ANOVA followed by multiple-comparison test.

Following the behavioral tests, body weight was measured, and brain tissues were collected for brain weight analysis. No significant differences in body weight were observed between vehicle- and PAV-615-treated male or female 2R and 149R mice (Figure 2A,B). Brain weight was significantly reduced in both vehicle and PAV-615 treated 149R mice compared to the treated 2R control mice (Figure 2C). PAV-615 treatment did not mitigate the reduction in brain weight in 149R mice compared to vehicle-treated 149R controls (Figure 2C). Together, these findings indicate that PAV-615 treatment has no significant effect on body or brain weight in 149R mice.

### 3.3. PAV-615 Mitigates DPR, TDP-43 and Stress Granule Pathologies in the 149R ALS/FTD Mouse Model

**Figure 3:**
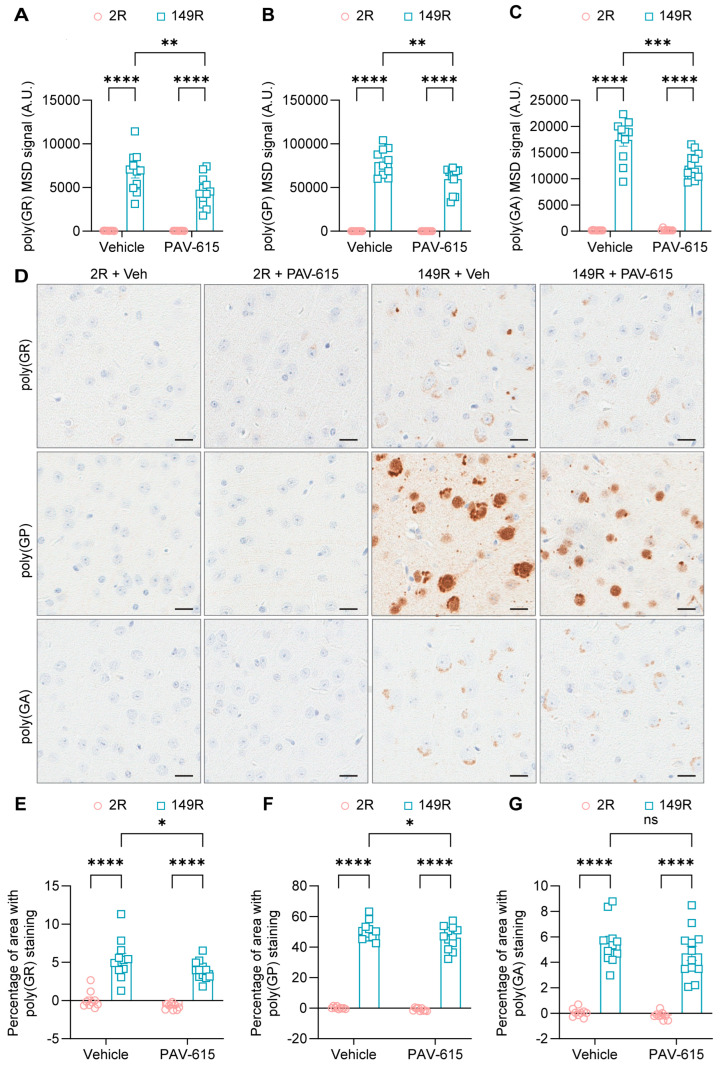
PAV-615 treatment reduces DPR proteins in the 149R ALS/FTD mouse model. (**A**–**C**) Quantification of poly(GR), poly(GP), and poly(GA) DPR proteins using MSD immunoassays in 6-month-old 2R and 149R mice treated with vehicle or PAV-615 (n = 9–12 per group). (**D**) Representative IHC images of poly(GR), poly(GP), and poly(GA) DPR proteins in the cortex of vehicle- and PAV-615-treated 2R and 149R mice. Scale bars, 20 μm. (**E**–**G**) Quantitative analysis of the DPR burden by measuring the percentage of cortical area with DPR pathology in vehicle- and PAV-615-treated 2R and 149R mice (n = 9–12 per group). Data shown as the mean ± SEM. ns: not significant, * *p* < 0.05, ** *p* < 0.01, *** *p* < 0.001, **** *p* < 0.0001, two-way ANOVA followed by multiple-comparison test.

Given that PAV-615 treatment improved behavioral performance in 149R mice (Figure 1), brain tissues were collected for biochemical and immunochemical analyses to determine whether PAV-615 reduces c9ALS/FTD-associated pathological hallmarks, including DPR proteins. Using our in-house Meso Scale Discovery (MSD) based ELISA assays, robust expression of DPR proteins including poly(GR), poly(GP), and poly(GA) were detected in 149R mice compared to 2R control mice (Figure 3A–C). Notably, PAV-615 treatment significantly reduced the levels of all three DPR proteins in 149R mice compared to vehicle-treated controls (Figure 3A–C). Consistent with immunoassay results, immunohistology (IHC) staining and quantitative analysis revealed that poly(GR), poly(GP), and poly(GA) were significantly accumulated in 149R mice (Figure 3D–G). Treatment with PAV-615 resulted in a significant reduction in poly(GR) and poly(GP) levels in 149R mice compared to the vehicle-treated 149R mice (Figure 3D–F). PAV-615 treatment also led to a decrease in poly(GA) levels in 149R mice, although this reduction did not reach statistical significance (Figure 3D,G). Together, these findings indicate that PAV-615 treatment attenuates c9ALS/FTD-associated DPR pathology in 149R mice.

**Figure 4:**
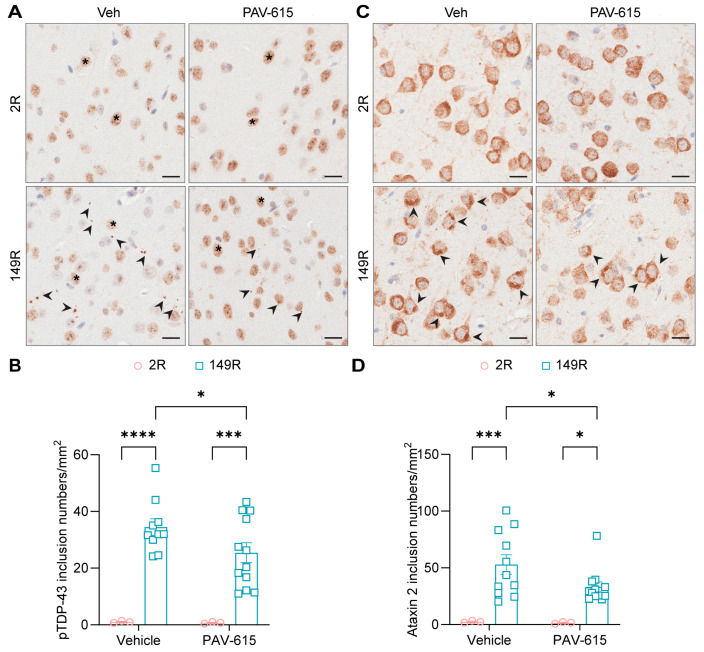
PAV-615 treatment reduces p-TDP43 and stress granules pathology in the 149R ALS/FTD mouse model. (**A**) Representative IHC images of pTDP-43 in the cortex of vehicle- and PAV-615-treated 2R and 149R mice. Black arrowheads indicate pTDP-43 inclusions; asterisks represent non-specific nuclear staining. Scale bars, 20 μm. (**B**) Quantitative analysis of the number of pTDP-43 inclusions per square millimeter of cortex in vehicle- and PAV-615-treated 2R and 149R mice (n = 3–12 per group). (**C**) Representative IHC images of ataxin 2 in the cortex of vehicle- and PAV-615-treated 2R and 149R mice. Black arrowheads indicate ataxin 2 inclusions. Scale bars, 20 μm. (**D**) Quantitative analysis of the number of ataxin 2-positive inclusions per square millimeter of cortex in vehicle- and PAV-615-treated 2R and 149R mice (n = 3–12 per group). Data shown as the mean ± SEM. * *p* < 0.05, *** *p* < 0.001, **** *p* < 0.0001, two-way ANOVA followed by multiple-comparison test.

Furthermore, given that inclusions of pTDP-43, another neuropathological hallmarks of c9ALS/FTD, have previously been observed in the brains of 149R mice [29], we investigated whether PAV-615 treatment could also reduce pTDP-43 pathology. IHC staining and quantitative analysis revealed that the number of pTDP-43 inclusions were significantly reduced in PAV-615-treated 149R mice compared to vehicle-treated 149R mice (Figure 4A,B). Additionally, because 149R mice develop inclusions positive for stress granule associated proteins, including G3BP1, ataxin 2, and eIF3η [29], we examined whether PAV-615 also attenuates stress granule pathology. PAV-615 treatment significantly reduced ataxin 2-positive inclusions compared to vehicle controls (Figure 4C,D). Together, these findings demonstrate that PAV-615 treatment reduces both TDP-43 and stress granule pathologies in 149R mice.

## 4. Discussion

In this study, we demonstrate that PAV-615, a novel THIQ-based assembly modulator, alleviates behavioral impairments in 149R mice, significantly improving motor performance while modestly reducing anxiety-like behavior and hyperactivity. PAV-615 treatment also significantly decreases c9ALS/FTD-related pathologies, including DPR protein accumulation, pTDP-43 inclusions, and ataxin 2-positive stress granules. Previous work demonstrated that THIQ-based assembly modulators rescue body weight loss and reduce neurofilament levels in SOD1-G93A ALS mice [28]. Our study investigated the neuroprotective effects of PAV-615, a novel THIQ-based assembly modulator, in a c9ALS/FTD mouse model. c9ALS/FTD patients exhibit pathological hallmarks of DPR and TDP-43, which are distinct from those observed in ALS patients carrying SOD1 mutations [13]. Notably, TDP-43 pathology is present in most ALS cases and in approximately 50% of FTD patients [12,13]. In combination of previous work [28], our results suggest that THIQ-based assembly modulators have broader therapeutic potential across ALS/FTD subtypes. Moreover, our findings highlight assembly modulator-mediated restoration of proteostasis as a promising strategy to mitigate pathological protein accumulation and improve functional outcomes in ALS/FTD. Additionally, our 149R mouse model robustly recapitulates the pathological hallmarks and behavioral deficits of c9ALS/FTD [29], and exhibits a response to antisense ASO treatment [31]. In line with these observations, our findings reinforce the value of this model as a preclinical platform for testing and advancing candidate therapeutic interventions.

**Figure 5:**
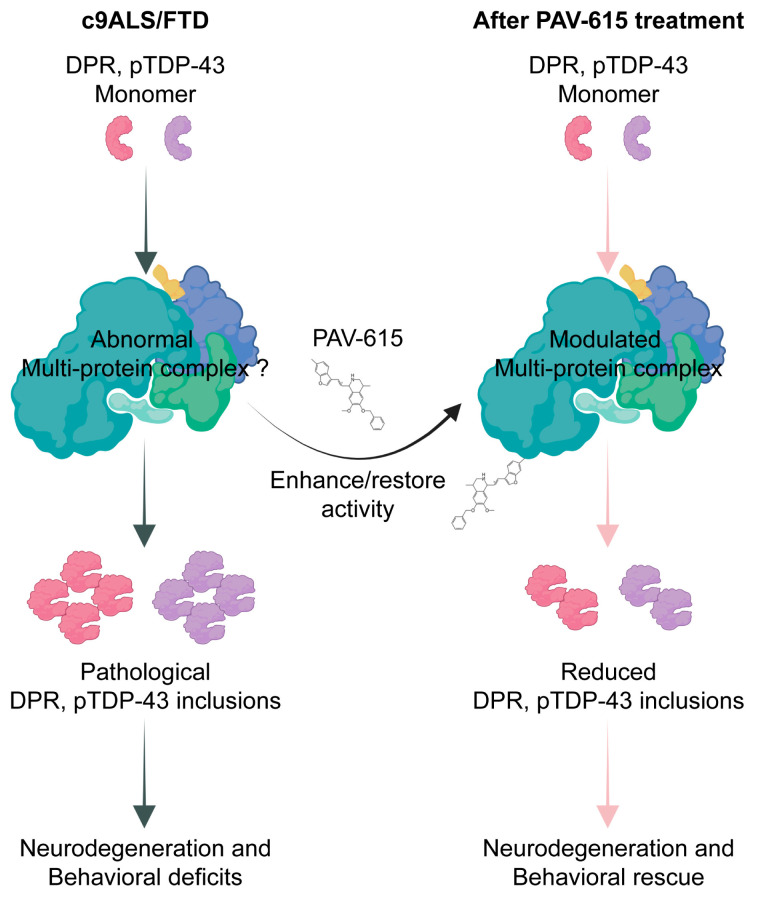
Potential mechanism of PAV-615 in reducing DPR and pTDP-43 pathologies and improving behavioral performance.

As a newly developed class of small-molecule protein assembly modulators, PAV-615 is proposed to restore cellular homeostasis by targeting transient, energy-dependent multiprotein complexes that regulate protein assembly and quality control. These compounds act allosterically, binding to non-catalytic sites and inducing conformational or compositional changes that enhance complex activity. In c9ALS/FTD, pathological DPR and pTDP-43 species may disrupt these complexes or impair their activity, thus leading to aggregation, cellular dysfunction and neurodegeneration. PAV-615 stabilizes the affected complex components, thereby restoring and/or enhancing their activity, ultimately reducing their pathological aggregation and preserving neuronal homeostasis (Figure 5).

To further elucidate the mechanism of action, it is essential to identify the specific multiprotein complex interacting with PAV-615, particularly the allosteric site that mediates direct compound binding. Previous studies using energy-dependent drug-resin affinity chromatography (eDRAC) followed by mass spectrometry identified proteins that interact with THIQ-based assembly modulators in both the brain tissue of SOD1-G93A transgenic mice and peripheral blood mononuclear cells (PBMCs) from ALS patients [28,32]. Of the 33 shared protein interactors reported across these studies, 26 belonged to the ALS protein interactome, including PDI, VCP, p62/SQSTM1, and RanGTPase [32]. Photocrosslinking experiments further demonstrated that PDI is a direct binding partner of THIQ compounds, consistent with its known role in allosteric regulation and disulfide-bond isomerization [33,34,35,36]. These findings suggest that PAV-615 may interact with PDI to restore and/or enhance the function of a disease-relevant multiprotein complex. Future investigations are needed to validate this hypothesis and define the specific regulatory targets and binding interface of PAV-615.

PAV-615 treatment provided beneficial effects but did not rescue the reduction in brain weight observed in 149R mice. Notably, our treatment paradigm began at 5 months of age, whereas disease-related pathologies in 149R mice have been reported as early as 3 months [29]. Thus, neurons could well have been irreversibly committed to death before onset of drug treatment. Future studies will investigate whether earlier intervention has greater efficacy in attenuating neuronal loss and pathological accumulation. In behavioral assays, PAV-615 modestly reduced anxiety-like behavior and hyperactivity in 149R mice. Although the trend toward reduced anxiety-like behavior approached statistical significance (*p* = 0.0963), the modest effect size may partly reflect the limited sample size. Expanding cohort sizes will be critical to fully assess the therapeutic potential of PAV-615 on behavioral phenotypes. Moreover, incorporating complementary assays, such as rotarod and grip strength tests, will provide a more comprehensive evaluation of motor coordination and neuromuscular function.

## 5. Conclusions

Our study demonstrates that the assembly modulator PAV-615 improves behavioral performance and reduces c9ALS/FTD-associated pathologies in a c9ALS/FTD mouse model, supporting the further development of assembly modulators as a therapeutic strategy for ALS/FTD.

## 6. Patents

The patent of PAV-615 resulting from the work reported in this manuscript is US12024521B2.

## Data Availability

The original contributions presented in this study are included in the article/Supplementary Materials. Further inquiries can be directed to the corresponding author.

## Supplementary Materials

The following supporting information can be downloaded at: https://www.mdpi.com/article/10.3390/cells14242012/s1, Figure S1: Synthesis of 6-benzyloxy-7-methoxy-4-methyl-1-[(E)-2-(6-methylbenzofuran-3-yl)vinyl]-1,2,3,4-tetrahydroisoquinoline (PAV-615); Figure S2: PAV-615 treatment modestly reduces hyperactivity in the 149R ALS/FTD mouse model; Table S1: Primary antibodies for immunohistochemistry staining; Table S2: Antibody information for MSD immunoassay. Ref. [37] is cited in the Supplementary Materials.

## Abbreviations

The following abbreviations are used in this manuscript:

AAV	Adeno-associated virus
ALS	Amyotrophic lateral sclerosis
BCA	Bicinchoninic Acid
*C9orf72*	Chromosome 9 open reading frame 72
CFPSA	Cell-free protein synthesis and assembly
DPR	Dipeptide repeat
eDRAC	Energy-dependent Drug Resin Affinity Chromatography
FTD	Frontotemporal dementia
G_4_C_2_	GGGGCC
IHC	Immunohistochemistry
IP	Intraperitoneal
MSD	Meso Scale Discovery
P0	Postnatal day 0
PBMC	Peripheral blood mononuclear cell
PDI	Protein disulfide isomerase
PK	pharmacokinetic
pTDP-43	Phosphorylated TAR DNA-binding protein 43
SOD1	Superoxide dismutase 1
TBSE	Tris-Buffered Saline with EDTA
THIQ	Tetrahydroisoquinolone
