# Supplementary material for: Preclinical Evaluation of the Assembly Modulator PAV-615 in a Mouse Model of C9orf72-Associated ALS/FTD
Source: Cells. 2025 Dec 17;14(24):2012. doi: 10.3390/cells14242012 (PMC12732003; doi:10.3390/cells14242012)
Supplement: Supplementary file 1 [file cells-14-02012-s001.zip › cells-3976675-supplementary.pdf]

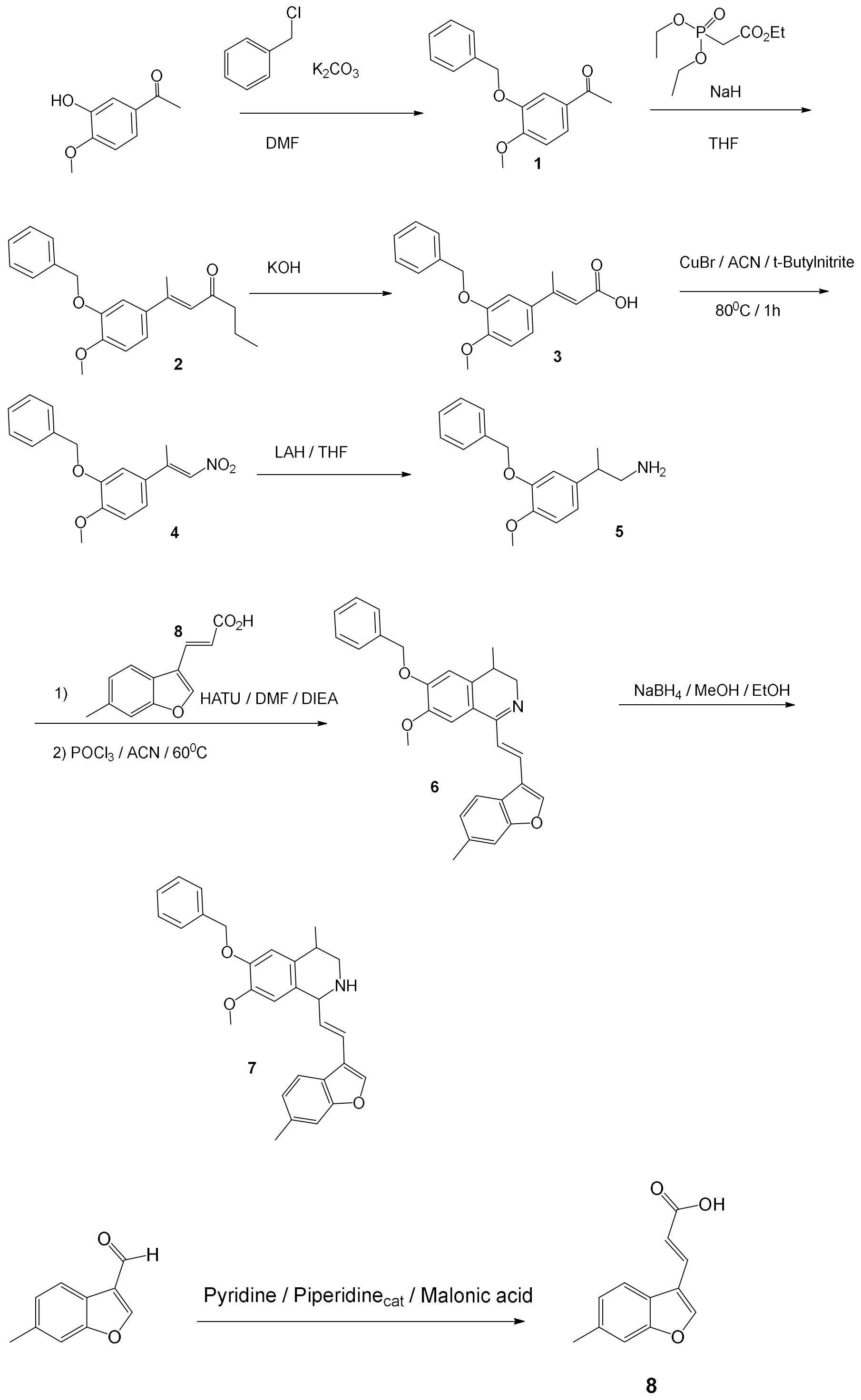

Supplementary Figure S1. Synthesis of 6-benzyloxy-7-methoxy-4-methyl-1-[(E)-2-(6-methylbenzofuran-3-yl)vinyl]-1,2,3,4-tetrahydroisoquinoline (PAV-615). PAV-615 was synthesized through a stepwise procedure involving the following intermediates: (**1**) 3 -Benzyloxy-4-methoxy-acetophenone; (**2**) 3-(3-Benzyloxy-4-methoxypheny-1)-2-butenoate; (**3**) 3-(3-Benzyloxy-4-methoxyphenyl)-2-butenoic acid; (**4**) 3-(3-Benzyloxy-4-methoxyphenyl)-1-nitro-2-butene; (**5**) 2-Methyl-2-(3-benzyloxy-4-methoxyphenyl)-l-aminoethane; (**6**) (E)-N-[2-(3-benzyloxy-4-methoxy-

phenyl)propyl]-3-(6-methylbenzofuran-3-yl)prop-2-enamide; (**7**) 6-benzyloxy-7-methoxy-4-methyl-1-[(E)-2-(6-methylbenzofuran-3-yl)vinyl]-1,2,3,4-tetrahydroisoquinoline; (**8**) (E)-3-(6-methyl1,3-benzodioxol-5-yl)-prop-2-enoic.

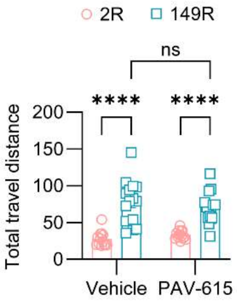

Supplementary Figure S2. PAV-615 treatment modestly reduces hyperactivity in the 149R ALS/FTD mouse model. Open field assay assessing hyperactivity by measuring the total travel distance in 6-month-old 2R and 149R mice treated with vehicle or compound (n = 13 – 15 per group). Data shown as the mean ± SEM. ns: not significant, **** $P < 0.0001$, two-way ANOVA followed by multiple-comparison test.

Table S1. Primary antibodies for immunohistochemistry staining

Antibody	Species	Dilution	Number
Anti-GR	Rabbit	1:2500	Rb7810[1]
Anti-GA	Rabbit	1:50000	Rb9880[1]
Anti-GP	Rabbit	1:10000	Rb5823[1]
Anti-pTDP-43	Rabbit	1:1000	Rb3655[2]

[1] Antibody described in: T. F. Gendron et al., Acta Neuropathol 126, 829-844 (2013).

[2] Antibody described in: J. Chew et al., Science 348, 1151-1154 (2015).

Table S2. Antibody information for MSD immunoassay

DPR	Plate	Capture antibody	Final capture antibody concentration	Detection antibody	Final detection antibody concentration	Mass of proteins per well	Dilution Buffer
Poly(GR)	96-well 1-Spot Sector Plates (MSD, #L15XA-3)	Anti-GR (Millipore, #MABN778, Rat)	2 µg/mL	Anti-GR (Proteintech, #23978-1-AP, Rabbit) + Sulfo-tagged Goat Anti Rabbit (MSD, #R32AB-1)	2 µg/mL	20 µg	Diluent 100
Poly(GA)	96-well 1-Spot Sector Plates (MSD, #L15XA-3)	Anti-GA (Millipore, #MABN889, Mouse)	2 µg/mL	Sulfo-tagged Anti-GA (Millipore, #MABN889, Mouse)	2 µg/mL	15 µg	TBS
Poly(GP)	96-well MSD GOLD Small spot streptavidin plate (MSD, #L15XA-3)	Biotinylated Anti-GP (Covance, #A1-0757)	2 µg/mL	Sulfo-tagged Anti-GP (Covance, #A1-0757)	2 µg/mL	15 µg	TBS
